# Comparison of the Efficacy of Glucagon-Like Peptide-1 Receptor Agonists in Patients With Metabolic Associated Fatty Liver Disease: Updated Systematic Review and Meta-Analysis

**DOI:** 10.3389/fendo.2020.622589

**Published:** 2021-02-16

**Authors:** Yuzhao Dai, He He, Sheyu Li, Lidan Yang, Xia Wang, Zhi Liu, Zhenmei An

**Affiliations:** ^1^Department of Endocrinology and Metabolism, West China Hospital, Sichuan University, Chengdu, China; ^2^Department of Laboratory Medicine, West China Hospital, Sichuan University, Chengdu, China

**Keywords:** metabolic associated fatty liver disease, non-alcoholic fatty liver disease (NAFLD), metabolic syndrome, systematic review, meta-analysis, glucagon-like peptide-1 receptor agonists (GLP-1 RAs)

## Abstract

**Aims:**

Metabolic associated fatty liver disease (MAFLD) is the most common cause of chronic liver disease and is a major health and economic burden in society. New drugs are urgently needed to treat MAFLD. This systematic review and meta-analysis was conducted to evaluate the efficacy of glucagon-like peptide-1 receptor agonists (GLP-1RAs) in patients with MAFLD.

**Method:**

We searched PubMed, Embase, Cochrane Library database, and Web of Science since 1977. We selected all randomized controlled trials which met the inclusion and exclusion criteria and evaluated the quality of evidence. A random-effects meta-analysis was performed to assess all the primary and second outcomes.

**Results:**

Eight randomized controlled trials, including 396 patients, of which 265 patients had type 2 diabetes mellitus, met the inclusion criteria. Compared with the placebo or active agents group, the GLP-RA group showed a significant reduction in the liver fat content [weight mean difference (WMD) -3.17%, 95%CI -5.30 to -1.03, P < 0.0001], body weight (WMD -4.58 kg, 95%CI -8.07 to -1.10, P = 0.010), waist circumference (WMD -3.74 cm, 95%CI -6.73 to -0.74, P = 0.010), alanine aminotransferase (WMD -10.73 U/L, 95%CI -20.94 to -0.52, P = 0.04), γ- glutamyl transferase (WMD -12.25 U/L,95% -18.85 to -5.66, P = 0.0003, with *I²*=23%), fasting blood glucose (MD, -0.36 mmol/L; 95%CI, -0.69 to -0.03, P = 0.030), and hemoglobin A1c (WMD -0.36%, 95%CI -0.52 to -0.19, P < 0.0001). The reported adverse events were gastrointestinal complications with no serious adverse events, and most symptoms were relieved within 1–2 weeks after dose titration.

**Conclusion:**

GLP-RAs may improve liver injury and metabolic disorder in patients with MAFLD, regardless of the presence of type 2 diabetes mellitus. The benefits of GLP-RAs treatment outweigh the adverse effects of drugs in patients with MAFLD.

## Introduction

Metabolic associated fatty liver disease (MAFLD) is the most common cause of chronic liver disease worldwide a common factor resulting in the requirement for liver transplantation to treat end-stage liver disease (1). According to a recent study, MALFD affects about a quarter of the world’s adult population, creating a major health and economic burden on society (2). In April of 2020, experts from 22 countries reached a consensus to define the diagnostic criteria for MAFLD based on the possible causes of the disease. The criteria are based on evidence of hepatic steatosis, in addition to one of the following three criteria, overweight/obesity, type 2 diabetes mellitus, or evidence of metabolic dysregulation (3). MAFLD exhibits a wide spectrum of histologic abnormalities ranging from hepatic steatosis to nonalcoholic steatohepatitis (NASH), which may progress to cirrhosis and even hepatocellular carcinoma.

The incidence of MAFLD is increasing along with its common comorbidities, including type 2 diabetes, obesity, dyslipidemia, and hyperuricemia (4–6). The first-line treatment is lifestyle intervention (7), with weight loss of 5%–10% beneficial for patients with MAFLD (8). However, most patients do not achieve or maintain dietary goals or their ideal body weight. In a meta-analysis of the treatment of MAFLD, more than 50% of patients failed to achieve their weight loss targets (9). Additionally, no pharmacotherapy for MAFLD has been approved. Many researchers have explored the drug treatment of MAFLD and found that various agents can help relieve the disease progression, such as thiazolidinone, vitamin E (PIVENS trial) (10), bile acid, obeticholic acid (FLINT trial) (11), and lipid-lowering drugs and other antioxidants. The differences between two or three therapies have also been evaluated (10).

It has been suggested that patients with MAFLD have lower concentrations of biologically active incretin hormones compared to healthy individuals (12), which may results from increased degradation by dipeptidyl peptidase-4 or a decreased production of the incretin hormones (13). GLP-1 can increase insulin synthesis and secretion in a glucose-dependent manner, and GLP-1 receptors have been found in various tissues of the human body (14). Recent animal studies confirmed that GLP-1RAs, in addition to weight loss and hypoglycemic effects, can reduce liver inflammatory lesions and even slow the process of steatosis change into fibrosis. As a newly hypoglycemic drug, researchers have observed that GLP-1RA can improve liver function and lipid metabolism in patients with diabetes showing elevated liver enzymes (15). GLP-1RAs suppress glucagon release from pancreatic alpha cells, delay gastric emptying, and enhance satiety. GLP-RAs can improve metabolic dysfunction, insulin resistance and lipotoxicity in key metabolic organs in the pathogenesis of MAFLD (16). Insulin resistance and lipotoxicity are pathognomonic features of MAFLD. Studies have shown that one of the diagnostic criteria for MAFLD is the presence of metabolic syndrome (3). Liraglutide, a GLP-1RA, has significant effects on glycemic control and can be used as a sub-treatment for patients with obesity with metabolic disorders to induce weight loss and insulin sensitivity (8). Various doses of GLP-1RAs have been used, and several randomized controlled trials (RCTs) have assessed the efficacy and safety of GLP-1RAs with other active agents or placebo.

A meta-analysis of the LEAD program (17) demonstrated that 26 weeks of liraglutide (1.8 mg) is safe, well-tolerated, and improves liver enzymes in patients with type 2 diabetes. This effect appears to be mediated by its action on weight loss and glycemic control. Meta-analysis is an essential method for estimating the comparative effectiveness of different treatments; therefore, we performed meta-analysis of studies conducted in the last 10 years to evaluate the effectiveness of GLP-1RAs for treating MAFLD.

## Material and Methods

The protocol for this systematic review was registered with PROSPERO (CRD42020187053). This systematic review and meta-analysis were conducted following the PRISMA guidelines.

### Search Strategy

Our search strategy was consist of entry terms and MeSH terms. We searched the Cochrane Library, Embase, PubMed and Web of Science for relevant articles published through April 20, 2020. We set no restrictions on the language of the articles. Clinical trial registry websites and conference abstracts were included in our search strategy, and we scanned the reference lists of eligible articles for additional eligible studies.

For example, in PubMed, we searched (“Non-alcoholic Fatty Liver Disease” OR “Fatty Liver*” OR “Nonalcoholic Fatty Liver Disease” OR “Nonalcoholic Fatty Liver*” OR “Nonalcoholic steatohepatitis*” OR “Non-alcoholic steatohepatitis” OR “NASH”) AND (“Glucagon-Like Peptide 1” OR “GLP-1” OR “Liraglutide” OR “Albiglutide” OR “Dulaglutide” OR “Semaglutide” OR “Lixisenatide” OR “Taspoglutide” OR “Exenatide” OR “Elsiglutide” OR “Teduglutide”). Eligible records were limited to RCTs. The details of the search strategy was shown in the supplementary material.

### Inclusion Criteria and Exclusion Criteria

Studies were considered as eligible if they met the following inclusion criteria. First, the populations were adult patients(aged>18 years) with MAFLD, with or without diabetes. MAFLD was diagnosed based on liver histology biopsy or imaging examination (ultrasonography, computed tomography, magnetic resonance imaging, magnetic resonance spectroscopy, and others). Included patients satisfied the following criteria: 1) alcohol intake not exceeding 21 standard drinks per week in men and 14 standard drinks per week in women over a 2‐year period preceding baseline liver histology or imaging examination (10); and 2) exclusion of secondary causes of liver diseases (e.g., autoimmune, viral hepatitis, Wilson’s disease or exposure to drugs that could induce steatosis). Second, the treatment intervention was GLP-1RAs (with no restriction on the GLP-RAs types). Third, the primary outcome was reduced severity of MAFLD or reduction in the liver fat fraction (LFF) or liver fat content (LFC) from baseline. The secondary outcomes were the change in body weight, waist circumference, liver enzyme levels [alanine aminotransferase (ALT), aspartate aminotransferase (AST), and/or γ-glutamyl transferase(γ-GGT)], and fasting blood glucose (FBG) from baseline. Fourth, the study design must be an RCT.

We excluded case reports, case series, cross-sectional, retrospective studies and observational studies. We also excluded conference abstracts for which full-text or complete data were not available.

### Studies Selection and Data Extraction

Trials from the database were managed using EndNote X9 software to remove duplicate articles. The titles and abstracts were independently screened by two reviewers (YD and ZL) and then independently screened the full-text articles. If any discrepancy between the two reviewers was found, the article would be resolved after discussion.

Two reviewers (YD and ZL) independently extracted the following information from eligible studies: study characteristics (first author, year of publication, sample size, intervention, the comparison group, follow-up time and patient age composition), and clinical outcomes (LFF/LFC, body weight, waist circumference, ALT, AST, γ-GGT, and FBG). All data were recorded in standard forms. Then the extraction results were checked by another reviewer (LY) and discussed to resolve any disagreements.

### Assessment of Risk of Bias of Included Studies

The quality of eligible studies was assessed with Cochrane Risk of Bias Tools (18), and included the following step: 1) random sequence generation (selection bias); 2) allocation concealment (selection bias); 3) blinding of participants and personnel (performance bias); 4) blinding of outcome assessment (detection bias); 5) incomplete outcome data (attrition bias); 6) selective reporting (reporting bias); 7) other bias; there were some biases that were not mentioned, which were closely related to the results. Each study was considered to have a “high risk of bias”, “low risk of bias”, or “unclear risk of bias”. Two authors (YD and ZL) independently assessed and checked the quality evaluation of RCTs. Any disagreement was discussed among these researchers or judged by another researcher (LY).

### Statistical Analysis

All statistical analyses were performed using Review Manager version 5.4.1. A Chi-square test was used to determine heterogeneity across studies (α=0.1). The *I^2^* statistic was applied to quantitatively evaluate the heterogeneity of studies. Studies with an *I^2^* statistic of 25%–50% were considered to have low heterogeneity, those with an *I^2^* of 50%–75%were considered to have moderate heterogeneity and those with an *I^2^* > 75% were considered to have high heterogeneity. A random effects model was applied regardless of heterogeneity. According to the characteristics of the studies, we conducted subgroup analyses and sensitivity analyses to explain the possible source of heterogeneity. Normally distributed continuous variables are described as the mean ± standard deviation. In some studies, the levels of changes in the outcomes were not reported. We requested this information from the corresponding author or used the conversion formulas recommended in the Cochrane Handbook Version 5.0.2 to calculate the changes in outcomes. Differences were expressed as the weight mean difference (WMD) with the 95% confidence interval (CI) for continuous outcomes. Results were considered as significant when P < 0.05.

## Results

### Study Flow and Characteristic of Studies

**Figure 1:**
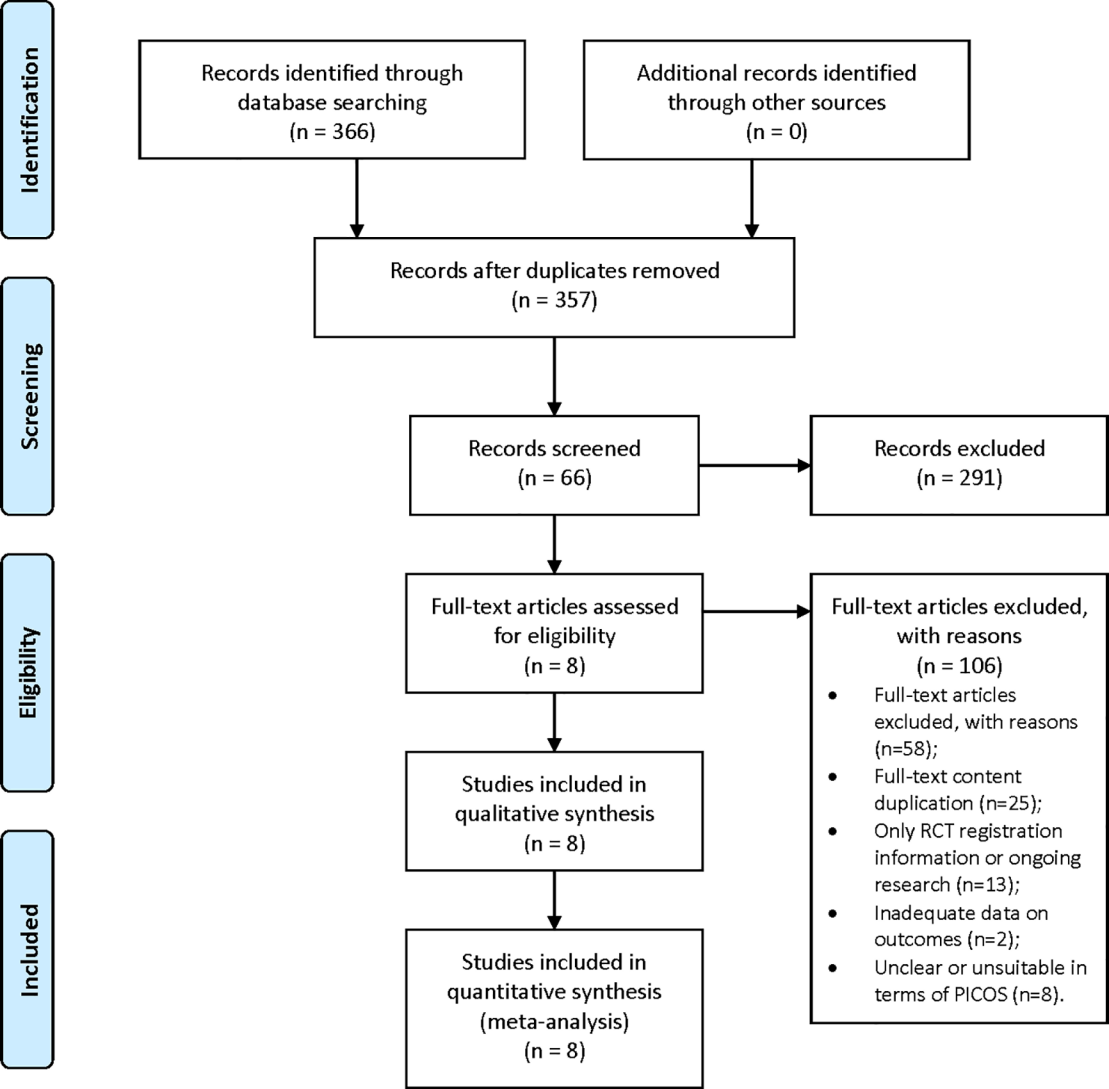
Flow diagram of study selection.

Through database searches, 366 records (PubMed 56; Embase 92; Cochrane Library 105; Web of Science 113) were found. After removing duplicates, 357 records remained; 66 records were selected for full-text assessment after screening the title and abstract, and 58 studies were excluded for the following reasons: 1) full-text content duplication (n=25); 2) only RCT registration information or trial in progress (n=13); 3) inadequate data on outcomes of interest (n=12); and 4) unclear or unsuitable in terms of PICOS (n=8). Only eight full-text articles, involving a number of 396 adult patients met the inclusion. The flow chart is shown in **Figure 1**.

The RCTs were published from 2014 to 2020 and the characteristics of the eight included studies are shown in **Table 1**. The 396 adult patients were involved in 6 trials of liraglutide (265 patients) and 2 trials of exenatide (131 patients). In these studies, fatty liver was diagnosed by either biopsy confirmation or imaging examination. Only Armstrong et al. (19) used histology biopsy to estimate the severity of MAFLD. We found that methods for non-invasively assessing liver fat content varied by study, with magnetic resonance imaging (MRI) used by Khoo (21, 22) and Yan (25), whereas Zhang and Liu (23, 26) used ^1^H-MRS. Shao (24) and Feng (20) used ultrasound imaging to assess liver fat content and stiffness. In addition, in control group, one study used liraglutide-placebo, one study used gliclazide, two studies used lifestyle modification, three studies used insulin therapy (both intensive insulin therapy or insulin plus metformin) and one study used pioglitazone plus metformin. A summary of the approaches used for liver steatosis assessment is shown in **Table 2**.

**Table 1:** Characteristics of included studies.

Study/Year	Sample (N)	T2DM (Y/N)	Age (year)	Intervention (Dose)		Follow-up time (Week)	Diagnostic method
				Experimental group	Control group		
Armstrong et al. (19)	52	Y	51.0 (11.4)	Liraglutide (max 1.8 mg qd)	Liraglutide-placebo (1.8 mg qd)	48	Biopsy-confirm
Feng et al. (20)	58	Y	47.4 (2.2)	Liraglutide (max 1.8 mg qd)	Gliclazide (max 120 mg qd)	24	USG
Khoo et al. (21)	24	N	41.4 (9.4)	Liraglutide (max 3 mg qd)	Lifestyle modification	26	MRI
Khoo et al. (22)	30	N	40.7 (9.1)	Liraglutide (max 3 mg qd)	Lifestyle modification	26	MRI
Liu et al. (23)	71	Y	49.1 (11.0)	Exenatide (max 10 μg bid)	Insulin glargine	24	^1^H-MRS
Shao et al. (24)	60	Y	43.0 (4.1)	Exenatide (max 10 μg bid) + insulin glargine	Intensive insulin therapy (insulin aspartate + insulin glargine)	12	USG
Yan et al. (25)	48	Y	44.4 (8.7)	Liraglutide (max 1.8 mg qd) +metformin 1.5 g	Insulin glargine + metformin 1.5 g	26	^1^H-MRS
Zhang et al. (26)	60	Y	50.9 (11.7)	Liraglutide (max 1.2mg qd) + metformin 0.5g tid	Pioglitazone (max 30 mg qd) + metformin 0.5 g tid	24	^1^H-MRS

USG, ultrasonography; MRI, magnetic resonance imaging; ^1^H-MRS, magnetic resonance spectroscopy.

**Table 2:** Summary of technology of liver steatosis assessment.

Study/Year	Sample (N)	Diagnostic method	Non-invasive tests	Cut-off value of inclusion criteria
Armstrong et al. (19)	52	Biopsy-confirm	N	Macrovesicular steatosis >5%, hepatocyte ballooning and lobular inflammation.
Feng et al. (20)	58	Quantitative ultrasonography	Y	Intrahepatic fat (IHF) ≥10% (measured by standardized ultrasonography H/R ratio and hepatic attenuation rate)
Khoo et al. (21)	24	MRI	Y	Liver fat fraction (LFF) ≥5.5% (measured with MRI in a predetermined standardized area of the liver)
Khoo et al. (22)	30	MRI	Y	Liver fat fraction (LFF) ≥5.5% (measured with MRI in a predetermined standardized area of the liver)
Liu et al. (23)	71	H-MRS	Y	Liver fat content (LFC) >10%
Shao et al. (24)	60	Abdominal ultrasound	Y	Fatty liver (FL) was qualitatively classified as absent FL, mild FL, moderate FL and severe FL
Yan et al. (25)	48	1H-MRS	Y	Intrahepatic lipid (IHL) >10% (measured by magnetic resonance imaging–estimated proton density fat fraction, MRI-PDFF)
Zhang et al. (26)	60	1H-MRS	Y	Hepatic fat content was measured by proton 1H-MRS on a 1.5 T whole-body MRI scanner

### Quality Assessment of the Evidence

**Figure 2:**
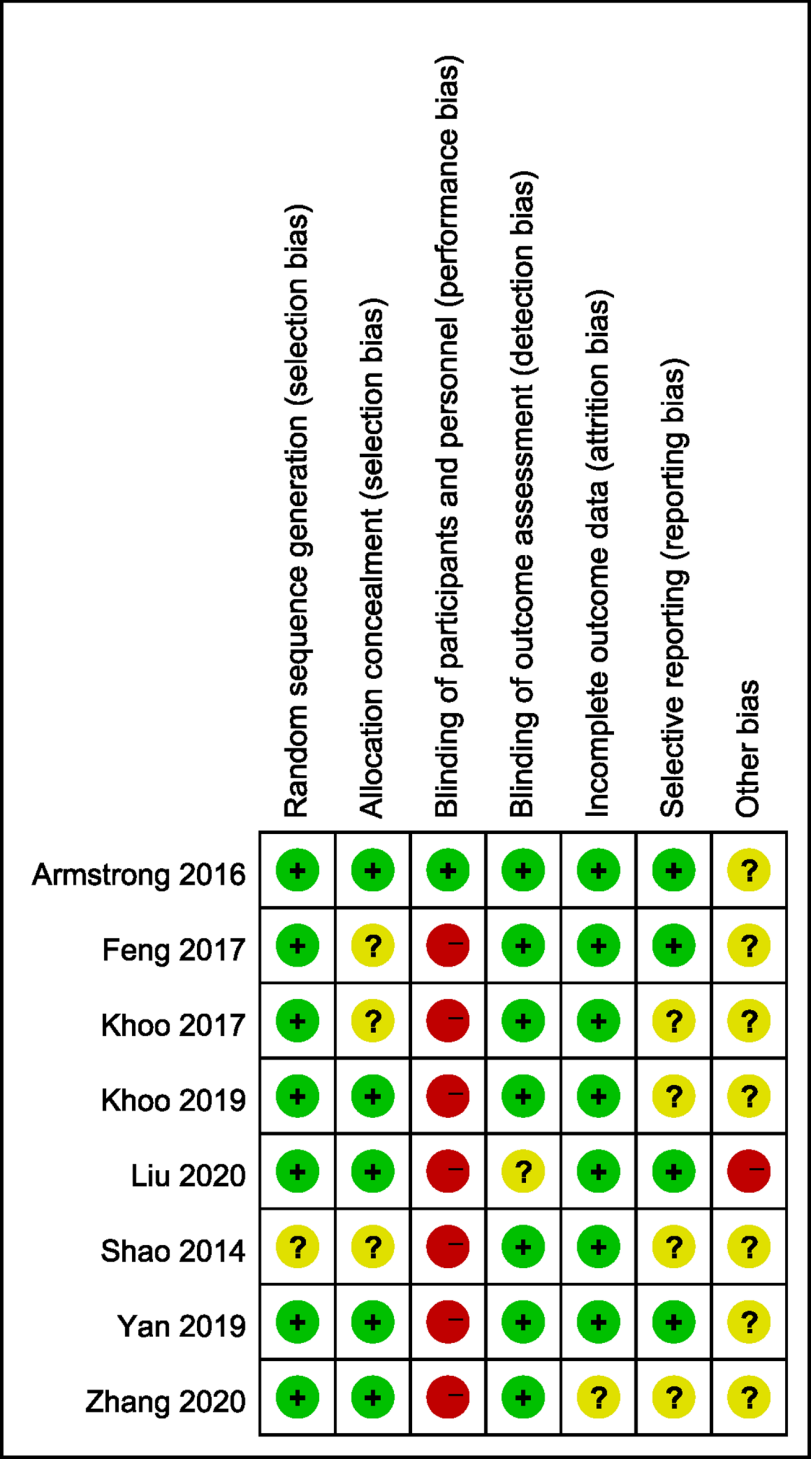
Risk of bias summary: review authors’ judgements about each risk of bias item for each included study.

**Figure 3:**
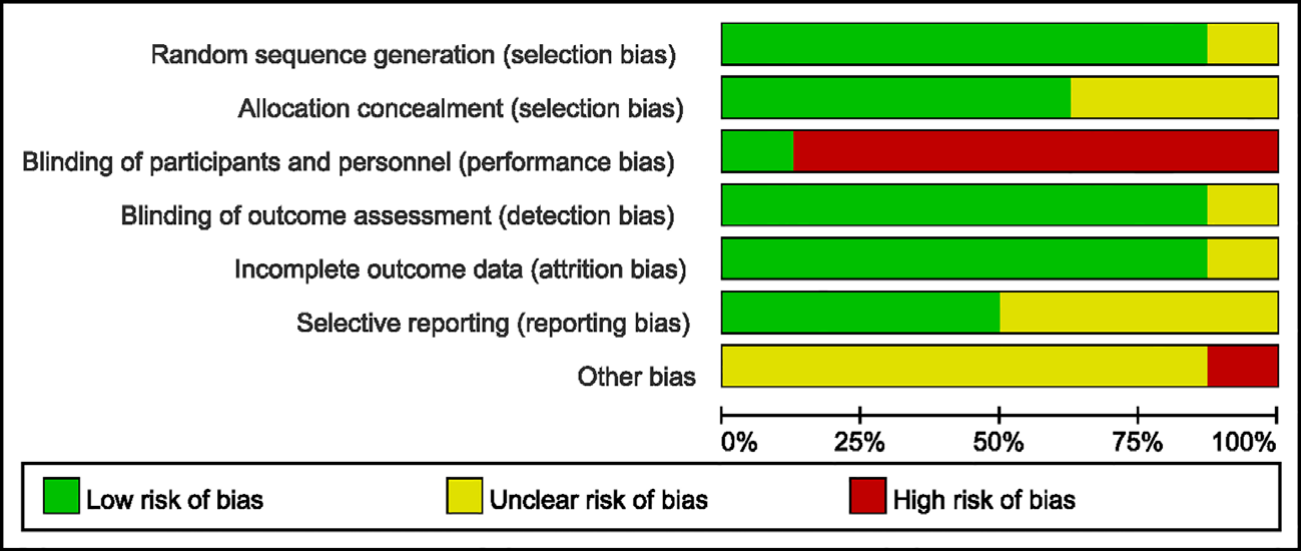
Risk of bias graph: review authors’ judgements about each risk of bias item presented as percentages across all included studies.

We evaluated the risk of bias of the included studies using Cochrane Risk of Bias Tools. We found that only the study by Armstrong et al. (19) had a low risk of bias. Seven of the eight studies lacked participant blinding, which means they were open-label RCTs. No participants drop out in three studies, whereas participants were lost to follow-up in five studies. Only one study did not report the reason for participant drop out and the solution to lost follow-up data. Based on these limitations, the quality of evidence for our assessment of the combined effects was downgraded. For the remaining bias, most studies were moderate or low risk and the details are shown in **Figures 2** and **3**.

### Effects of Interventions

The included eight studies consisted of six liraglutide therapy studies and two exenatide therapy studies. Subgroup analysis was conducted when necessary.

### Primary Outcome

Only one study used biopsy to measure improvement in liver fibrosis and steatosis from baseline to the end of treatment [Armstrong et al. (19)]. In this trial, 9 (39%) of 23 patients in the liraglutide group shown resolution of definite non-alcoholic steatohepatitis compared with 2 (2%) of 22 patients in the placebo group (RR, 4.3; 95%CI, 1.0 to 17.7; P =0.019). Fewer patients in the liraglutide group exhibited progression of fibrosis compared with placebo group and a greater proportion of patients in the liraglutide group showed improvement in steatosis and hepatocyte ballooning compared with the placebo group.

**Figure 4:**
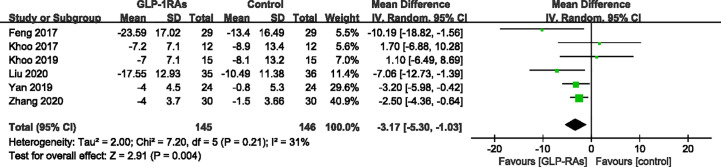
Mean difference or standard mean difference in change from baseline between glucagon-like peptide-1 receptor agonists (GLP-1RAs) vs. control. SD, Standard deviation; CI, confidence interval; IV, inverse variance; liver fat fraction or liver fat content (%).

We conducted a meta-analysis of LFC or LFF among 6 studies (20–23, 25, 26), which included 291 participants. A heterogeneity test showed that the result had low heterogeneity, with *I^2^* = 31%. GLP-RAs significantly improved the LFC (WMD -3.17%, 95%CI -5.30 to -1.03, P < 0.0001) (**Figure 4**). Shao et al. (24) used ultrasound to divide the LFC into four levels: absent, mild, moderate, and severe. They measured degrees of severity of fatty liver (FL) from baseline to the end of treatment in the exenatide and intensive insulin therapy groups. After the study, they observed a regression from a greater to lower degree of FL in both groups. The reversal rate in the exenatide group was 93.3% versus 66.7% in the intensive insulin therapy group, with a significant difference between groups (P < 0.01). Although the methods used in each study varied, GLP-RAs groups were more likely to demonstrate significant improvements in LFC compared to the control groups.

### Secondary Outcomes

#### Effect of GLP-RAs on Anthropometric Measuring

**Figure 5:**
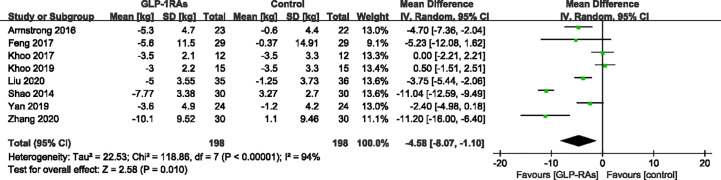
Mean difference or standard mean difference in change from baseline between glucagon-like peptide-1 receptor agonists (GLP-1RAs) vs. control. SD, Standard deviation; CI, confidence interval; IV, inverse variance; body weight (kg).

**Figure 6:**
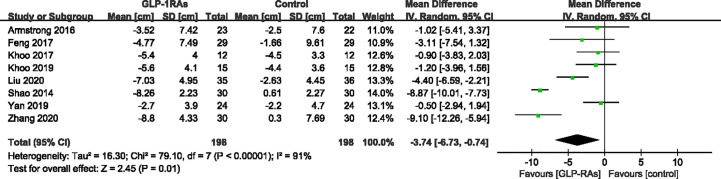
Mean difference or standard mean difference in change from baseline between glucagon-like peptide-1 receptor agonists (GLP-1RAs) vs. control. SD, Standard deviation; CI, confidence interval; IV, inverse variance; waist circumference (cm).

All eight studies (396 patients) that met the requirements reported changes in body weight and waist circumference before and after treatment. For body weight, heterogeneity was measured as an *I²* of 94% (P < 0.00001). In contrast to the control group, GLP-RAs significantly reduced body weight (WMD -4.58kg, 95%CI -8.07 to -1.10, P=0.010) (**Figure 5**) compared with the control group. The measurements of waist circumference also shown high heterogeneity (*I²* = 91%). According to a random-effect model, there were significant differences in the GLP-1RA group to in compared with the control group (WMD -3.74 cm, 95%CI -6.73 to -0.74, P = 0.010) (**Figure 6**). We then performed subgroup analysis between liraglutide and exenatide treatment. Both liraglutide and exenatide therapy significantly reduced body weight (WMD, -3.25 kg; 95%CI, -6.73 to -0.74; P = 0.03 vs. WMD, -7.40 kg; 95%CI, -14.55 to -0.26; P = 0.04) and waist circumference (WMD, -2.61 cm; 95%CI, -5.35 to 0.13; P = 0.06 vs. WMD, -6.74 cm; 95%CI, -11.11 to -2.36; P = 0.003).

#### Effect of GLP-RAs on Serum Liver Enzyme

Eight studies reported the effects of GLP-RAs on ALT and AST. Particularly, Zhang (26) found that the statistics of ALT, AST, and γ-GGT had skewed distributions, and logarithmic transformation shown that the data had a lognormal distribution. Considering these differences in statistics distributions, we temporarily removed the data from the study by Zhang to evaluate the effects of ALT, AST, and γ-GGT levels.

**Figure 7:**
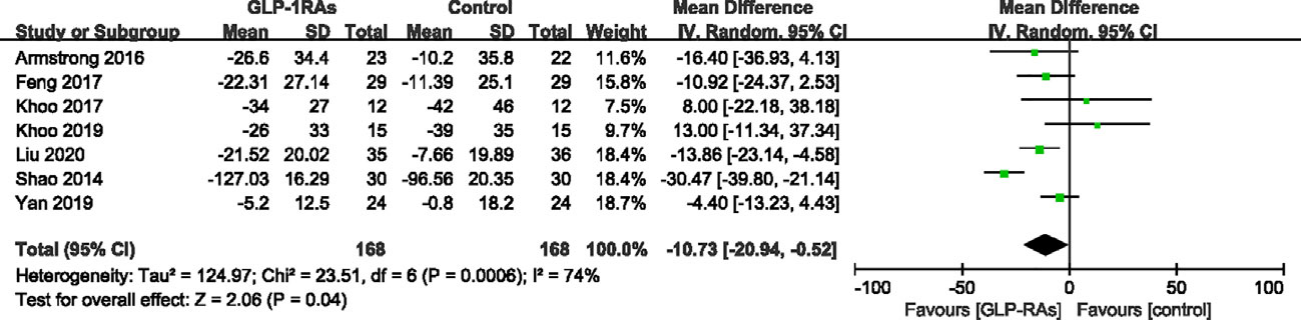
Mean difference or standard mean difference in change from baseline between glucagon-like peptide-1 receptor agonists (GLP-1RAs) vs. control. SD, Standard deviation; CI, confidence interval; IV, inverse variance; alanine transaminase (ALT).

**Figure 8:**
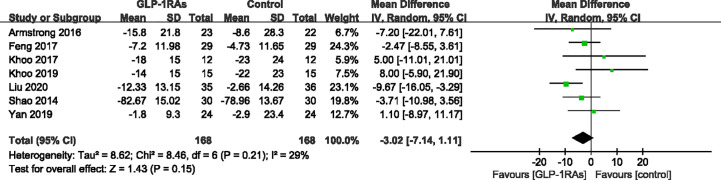
Mean difference or standard mean difference in change from baseline between glucagon-like peptide-1 receptor agonists (GLP-1RAs) vs. control. SD, Standard deviation; CI, confidence interval; IV, inverse variance; aspartate aminotransferase (AST, U/L).

**Figure 9:**
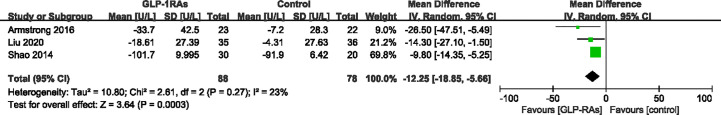
Mean difference or standard mean difference in change from baseline between glucagon-like peptide-1 receptor agonists (GLP-1RAs) vs. control. SD, Standard deviation; CI, confidence interval; IV, inverse variance; gamma-glutamyl transpeptidase (γ-GGT, U/L).

After treatment, ALT levels of the GLP-RAs groups were significantly decreased compared to those in the control group. The GLP-RAs improved ALT compared with the control group (WMD -10.73 U/L, 95%CI -20.94 to -0.52, P = 0.04), showing high heterogeneity (*I ²*= 74%) (**Figure 7**). Subgroup analysis of the different therapies revealed a significant difference in the reduction of ALT in the exenatide group (WMD -22.16 U/L,95%CI -38.44 to -5.88, P = 0.008, *I^2^* = 84%) compared to in the control group. However, this effect was not observed for the liraglutide group (WMD -5.21 U/L,95%CI -12.93 to 2.51, P = 0.19). We found that GLP-1RAs did not significantly affect AST compared with the control group (WMD -0.17 U/L, 95%CI -0.44 to 0.09, P = 0.15, *I²* = 29%) (**Figure 8**). Only four studies (19, 23, 24, 26) reported results for γ-GGT; we chose three of these studies to evaluate the combined effects. The random-effect model shown that GLP-1RAs had a significant effect on γ-GGT (WMD -12.25 U/L,95% -18.85 to -5.66, P = 0.0003, with *I²* = 23%) (**Figure 9**).

#### Effect of GLP-RAs on Glucose Metabolism

**Figure 10:**
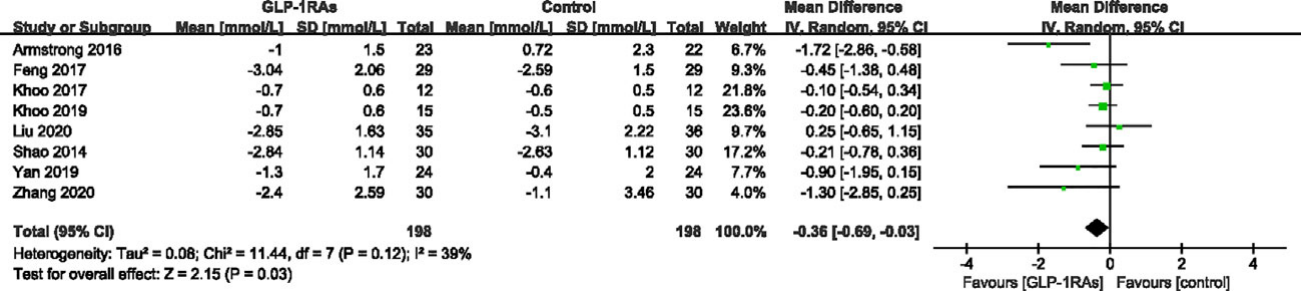
Mean difference or standard mean difference in change from baseline between glucagon-like peptide-1 receptor agonists (GLP-1RAs) vs. control. SD, Standard deviation; CI, confidence interval; IV, inverse variance; fasting blood-glucose (FBG, mmol/L).

**Figure 11:**
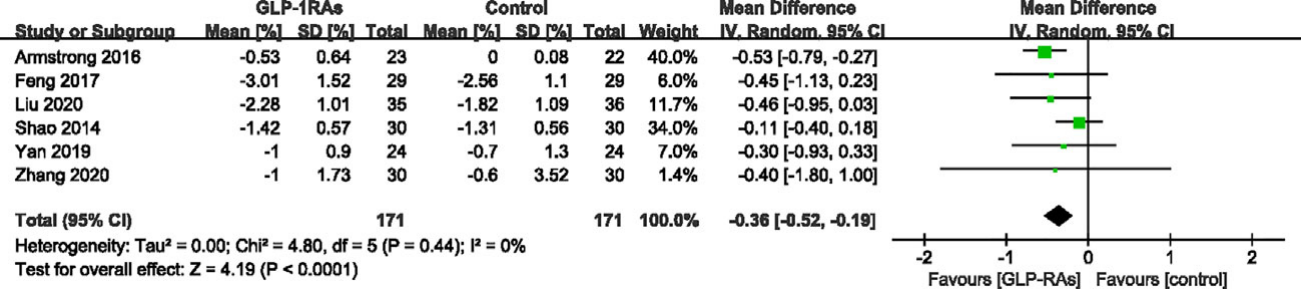
Mean difference or standard mean difference in change from baseline between glucagon-like peptide-1 receptor agonists (GLP-1RAs) vs. control. SD, Standard deviation; CI, confidence interval; IV, inverse variance; HbA1c (%).

All eight trials reported changes in FBG; the heterogeneity was 39%, which was assessed by *I²*. A random-effects model demonstrated a significant difference between the GLP-1RAs group and control group (WMD -0.36 mmol/L, 95%CI -0.69 to -0.03, P = 0.030) (**Figure 10**). HbA1c was reported in six trials (342 patients) with low heterogeneity (*I²*=0%). The meta-analysis showed a significant reduction in Hb1Ac in patients treated with GLP-1RAs compared to those in the control group (WMD -0.36%, 95%CI -0.52 to -0.19, P < 0.0001) (**Figure 11**).

### Safety

All studies recorded adverse events (AEs), which were mostly gastrointestinal complications with no serious AEs, such as serious hypoglycemia or acute pancreatitis. The main AEs involved nausea, vomiting, diarrhea, decreased appetite, flatulence, and abdominal pain. Adverse gastrointestinal reactions mainly occur during the dose increase period, and most AE symptoms are relieved within 1–2 weeks after dose titration. The incidence of adverse reactions was related to the drug dose used in the study. Particularly, in two studies by Khoo et al. (21, 22), patients were administered 3 mg liraglutide per day, increasing the incidence of adverse reactions.

### Sensitivity Analysis

We carried out sensitivity analysis to test for heterogeneity. Removing each article from the analysis individually, suggested that the study by Shao et al. was the source of heterogeneity. For ALT, after deleting the article, the *I^2^*-value changed from 74% to 28%, and the P-value changed slightly (from 0.03 to 0.04). While the effect of ALT levels changed from (WMD -10.73 U/L, 95%CI -20.94 to -0.52) to (WMD -7.69 U/L, 95%CI -14.57 to -0.80). We did not observe the same change in body weight or waist circumference. These changes may be related to the patients’ baseline ALT level, which was higher in the study by Shao et al. (patients with ALT >2.5-fold and <5-fold the upper limit of normal were included). However, most of these patients were excluded from other studies.

Based on the differences between control groups, we temporarily excluded the two studies by Khoo et al. because of the large clinical heterogeneity in the trial design of these studies. After removing these studies, we conducted the combined analysis again and found that the ALT level in the GLP-RAS group was significantly improved compared with that in the control group (WMD, -15.21; 95%CI, -25.45 to -4.98; P = 0.004). Other outcomes, such as AST, FBG, and HbA1c, showed low heterogeneity, and thus we did not perform further tests.

### Publication Bias

As the number of studies included was <10, we did not carry out a test of publication bias or draw funnel plots.

## Discussion

In our systematic review and meta-analysis, we evaluated the efficacy and safety of GLP-1 RAs for patients with MAFLD. However, only liraglutide and exenatide therapy were included in our assessment of the effects of MAFLD treatment. For meta-analysis of LFC or LFF among six studies, we used the fixed-effect model and standard mean differences, which showed that GLP-RAs significantly improved the LFC (WMD -3.17%, 95%CI -5.30 to -1.03, P < 0.0001).

Heterogeneity existed in the trial designs of the included studies, Khoo et al. mainly selected patients with MAFLD without diabetes. In the current consensus of MAFLD, type 2 diabetes is one of the three auxiliary diagnostic criteria (3). Therefore, there may be differences in the baseline characteristics and outcome indicators, such as FBG levels. Moreover, in Khoo et al.’s study, the maximum dose of liraglutide was 3 mg per day rather than the more common 1.8 mg per day. According to previous studies (19), the adverse effects of GLP-RAs were related to its dosage, and the authors also reported a higher probability of gastrointestinal adverse events. In contrast, large doses of liraglutide (3 mg per day) have been approved for weight management in obesity, and studies confirmed the dose-dependent weight loss effect (27). However, whether its direct effect on the liver is increased requires further analysis. Notably, in the study by Khoo et al. in 2019 (22), at the end of 26 weeks of intervention, the treatment effects were equivalent in the two groups. After stopping liraglutide and continuing follow-up for another 26 weeks, they found that the GLP-RAs group did not maintain the same level of improvement as the control group. This information may facilitate analysis of the optimal dosage and treatment course for GLP-RAs, and may help promote research on the after-effects of these new drugs.

The gold standard of the MAFLD diagnostic method is histological biopsy, which is an invasive method. Because of objective problems such as the difficulty of the puncture biopsy technique, difficulty in obtaining pathological samples, and patients’ refusal of invasive examination, many doctors tend to choose non-invasive tests (NITs) in clinical practice. As a result, researchers may choose NITs rather than pathological biopsy as the primary outcome indicator to estimate liver disease. In October, the Asia Pacific Association for the Study of the Liver (APASL) recommended the use of NITs for the diagnosis of MAFLD, disease severity assessment, disease progression and treatment response monitoring (28). However, liver biopsy remains the gold standard for the diagnosis and staging of steatohepatitis and fibrosis, particularly in patients with uncertain clinical manifestations, critical NIT results or inconsistent with clinical manifestations. Armstrong et al. conducted the first randomized controlled study to evaluate liver histology both at baseline and at the end of the study, ensuring that all patients included in the study were diagnosed using the gold-standard method for MAFLD. In this study, a greater proportion of patients administered liraglutide showed improvements in steatosis and hepatocyte ballooning (19). Liraglutide also improves weight and glycemic control may help improve the risk of future cardiovascular disease and premature death in patients with nonalcoholic steatohepatitis.

The efficient and safe drug for treating MAFLD is a complex problem, and there is no recommended drug treatment plan for MAFLD. Most treatment methods are based on weight loss, glucose metabolism improvement, and anti-oxidative stress methods, such as vitamin E, thiazolidinediones, statins, and dipeptidyl peptidase-4 inhibitors. Sanyal (10) designed a three-arm study showing that for non-diabetic patients with NASH, vitamin E and pioglitazone have a better effect on liver function, but the long-term effectiveness and safety require further research. Furthermore, GLP-1R expression has been identified in the hepatocytes of both rodents and humans (29). Previous studies suggested that the effects of GLP-1RAs on the liver cannot be fully explained by weight loss and hypoglycemic effects. It has been confirmed in animal studies that liraglutide can alleviate liver steatosis, insulin resistance and endoplasmic reticulum oxidative stress in mice without weight loss (30, 31). A recent animal study showed that liraglutide can modulate the expression and activity of the hepatic renin-angiotensin system through the GLP-1/RAS axis and ameliorate MAFLD (32). Research on GLP-1 in patients with liver injury has gradually increased; but is still mainly based on retrospective studies, case reports or cohort studies.

In the past decade, there have been some meta-analyses for GLP-RA treatment of MAFLD (33, 34). Dong et al. (33) evaluated three RCTs and three observational studies (a total of 329 people), among which one RCT and two observational trials used liver pathological biopsy as a diagnostic method. They found that GLP-RAs can significantly improve liver histological changes and liver enzyme changes in biopsy-confirmed patients with NASH. However, because of the small sample size of the meta-analysis, the reliability of the results requires further study. GLP-RAs, as a relatively safe hypoglycemic drug, reported that most AEs were almost gastrointestinal complications. These gastrointestinal AEs mainly occur during the dose increase period, and most AE symptoms are relieved within 1–2 weeks after dose titration.

Our study had several limitations. First, considering the wide clinical heterogeneity of different RCTs, such as different design methods, drug types, drug dosages and different choices of the control group, we used a random-effects model in some analyses to reduce the influence of these factors. We also excluded some conference abstracts, clinical trial registration information, and posters, because we did not have access to the full-text articles and data. Moreover, the search and screening process of registration information revealed several eligible ongoing studies (35–40) concentrating on the effects of different types of GLP-RAs, such as semaglutide and dulaglutide. In recent years, several observational trials, cohort studies, case reports, and one-arm studies (41), have demonstrated significant improvements in liver steatosis, serum liver enzyme, and glycolipid metabolism disorder.

## Conclusion

In summary, we demonstrated that GLP-1RAs reduce liver fat content and body weight and improve the laboratory metabolic parameters in adults with MAFLD without serious safety concerns. Thus, GLP-1RAs can be used as anti-MAFLD drugs, particularly in patients at a higher risk of MAFLD complications. Given the limited study population, randomized trials of observational cohorts are needed to confirm the clinical usage of GLP-1RAs in adults with MAFLD.

## Data Availability Statement

The original contributions presented in the study are included in the article/**Supplementary Material**. Further inquiries can be directed to the corresponding author.

## Author Contributions

Conception and design of the study: ZA, HH, SL, YD. Screened databases, extracted analysis the study data: LY, ZL, YD. Drafting of the initial manuscript: HH, YD. Verified the study methodology: SL, XW, LY. Revision of the manuscript: HH, SL, XW. All authors contributed to the article and approved the submitted version.

## Funding

This work was supported by the Science and Technology Department of Sichuan Province [grant NO. 2020YFS0096]; and the National Natural Science Foundation of China [Grant NO. 81902142].

## Conflict of Interest

The authors declare that the research was conducted in the absence of any commercial or financial relationships that could be construed as a potential conflict of interest.

## Abbreviations

MAFLD, metabolic associated fatty liver disease; HCC, hepatocellular carcinoma; GLP-1RAs, glucagon-like peptide-1 receptor agonists;DDP-4, dipeptidyl peptidase 4; RCTs, randomized controlled trials; T2DM, type 2 diabetes mellitus; MD, mean difference; CI, confidence interval; LFC, liver fat content; LFF, liver fat fraction; BMI, body mass index; ALT, alanine transaminase; AST, aspartate transaminase; γ-GGT, gamma-glutamyl transpeptidase; FBG, fasting blood-glucose; TC, total cholesterol; TG, total triglyceride; HDL-C, high-density lipoprotein cholesterol; LDL-C, low-density lipoprotein cholesterol; AEs, adverse events; SAEs, serious adverse events.

## References

[1] Armstrong MJ Adams LA Canbay A Syn WK. Extrahepatic complications of nonalcoholic fatty liver disease. Hepatology (2014) 59(3):1174–97. 10.1002/hep.26717 24002776

[2] Eslam M Sanyal AJ George J International Consensus P. MAFLD: A Consensus-Driven Proposed Nomenclature for Metabolic Associated Fatty Liver Disease. Gastroenterology (2020) 158(7):1999–2014 e1. 10.1053/j.gastro.2019.11.312 32044314

[3] Eslam M Newsome PN Sarin SK Anstee QM Targher G Romero-Gomez M. A new definition for metabolic dysfunction-associated fatty liver disease: An international expert consensus statement. J Hepatol (2020) 73(1):202–9. 10.1016/j.jhep.2020.07.045 32278004

[4] Li Q Li X Wang J Liu H Kwong JS Chen H. Diagnosis and treatment for hyperuricemia and gout: a systematic review of clinical practice guidelines and consensus statements. BMJ Open (2019) 9(8):e026677. 10.1136/bmjopen-2018-026677 PMC6720466 31446403

[5] Hou Q Yu C Li S Li Y Zhang R Zheng T. Characteristics of lipid profiles and lipid control in patients with diabetes in a tertiary hospital in Southwest China: an observational study based on electronic medical records. Lipids Health Dis (2019) 18(1):13. 10.1186/s12944-018-0945-8 30636643 PMC6330454

[6] Li S Yu C Li Y Li Q Zhang R Hou Q. Study design and baseline characteristics of inpatients with diabetes mellitus in a tertiary hospital in China: A database study based on electronic medical records. J Evid Based Med (2018) 11(3):152–7. 10.1111/jebm.12291 29512333

[7] Chalasani N Younossi Z Lavine JE Diehl AM Brunt EM Cusi K. The diagnosis and management of non-alcoholic fatty liver disease: practice Guideline by the American Association for the Study of Liver Diseases, American College of Gastroenterology, and the American Gastroenterological Association. Hepatology (2012) 55(6):2005–23. 10.1002/hep.25762 22488764

[8] Sberna AL Bouillet B Rouland A Brindisi MC Nguyen A Mouillot T. European Association for the Study of the Liver (EASL), European Association for the Study of Diabetes (EASD) and European Association for the Study of Obesity (EASO) clinical practice recommendations for the management of non-alcoholic fatty liver disease: evaluation of their application in people with Type 2 diabetes. Diabetes Med (2018) 35(3):368–75. 10.1111/dme.13565 29247558

[9] Musso G Gambino R Cassader M Pagano G. A meta-analysis of randomized trials for the treatment of nonalcoholic fatty liver disease. Hepatology (2010) 52(1):79–104. 10.1002/hep.23623 20578268

[10] Sanyal AJ Chalasani N Kowdley KV McCullough A Diehl AM Bass NM. Pioglitazone, vitamin E, or placebo for nonalcoholic steatohepatitis. N Engl J Med (2010) 362(18):1675–85. 10.1056/NEJMoa0907929 PMC2928471 20427778

[11] Neuschwander-Tetri BA Loomba R Sanyal AJ Lavine JE Van Natta ML Abdelmalek MF. Farnesoid X nuclear receptor ligand obeticholic acid for non-cirrhotic, non-alcoholic steatohepatitis (FLINT): a multicentre, randomised, placebo-controlled trial. Lancet (2015) 385(9972):956–65. 10.1016/S0140-6736(14)61933-4 PMC4447192 25468160

[12] Bernsmeier C Meyer-Gerspach AC Blaser LS Jeker L Steinert RE Heim MH. Glucose-induced glucagon-like Peptide 1 secretion is deficient in patients with non-alcoholic fatty liver disease. PloS One (2014) 9(1):e87488. 10.1371/journal.pone.0087488 24489924 PMC3906180

[13] Seghieri M Christensen AS Andersen A Solini A Knop FK Vilsboll T. Future Perspectives on GLP-1 Receptor Agonists and GLP-1/glucagon Receptor Co-agonists in the Treatment of NAFLD. Front Endocrinol (Lausanne) (2018) 9:649. 10.3389/fendo.2018.00649 30459715 PMC6232120

[14] Bifari F Manfrini R Dei Cas M Berra C Siano M Zuin M. Multiple target tissue effects of GLP-1 analogues on non-alcoholic fatty liver disease (NAFLD) and non-alcoholic steatohepatitis (NASH). Pharmacol Res (2018) 137:219–29. 10.1016/j.phrs.2018.09.025 30359962

[15] Fan H Pan Q Xu Y Yang X. Exenatide improves type 2 diabetes concomitant with non-alcoholic fatty liver disease. Arquivos Bras Endocrinol e Metabol (2013) 57(9):702–8. 10.1590/S0004-27302013000900005 24402015

[16] Armstrong MJ Hull D Guo K Barton D Hazlehurst JM Gathercole LL. Glucagon-like peptide 1 decreases lipotoxicity in non-alcoholic steatohepatitis. J Hepatol (2016) 64(2):399–408. 10.1016/j.jhep.2015.08.038 26394161 PMC4713865

[17] Armstrong MJ Houlihan DD Rowe IA Clausen WH Elbrønd B Gough SC. Safety and efficacy of liraglutide in patients with type 2 diabetes and elevated liver enzymes: individual patient data meta-analysis of the LEAD program. Aliment Pharmacol Ther (2013) 37(2):234–42. 10.1111/apt.12149 23163663

[18] Furlan AD Malmivaara A Chou R Maher CG Deyo RA Schoene M. 2015 Updated Method Guideline for Systematic Reviews in the Cochrane Back and Neck Group. Spine (Phila Pa 1976) (2015) 40(21):1660–73. 10.1097/BRS.0000000000001061 26208232

[19] Armstrong MJ Gaunt P Aithal GP Barton D Hull D Parker R. Liraglutide safety and efficacy in patients with non-alcoholic steatohepatitis (LEAN): a multicentre, double-blind, randomised, placebo-controlled phase 2 study. Lancet (london Engl) (2016) 387(10019):679–90. 10.1016/S0140-6736(15)00803-X 26608256

[20] Feng W Gao C Bi Y Wu M Li P Shen S. Randomized trial comparing the effects of gliclazide, liraglutide, and metformin on diabetes with non-alcoholic fatty liver disease. J Diabetes (2017) 9(8):800–9. 10.1111/1753-0407.12555 28332301

[21] Khoo J Hsiang J Taneja R Law NM Ang TL. Comparative effects of liraglutide 3 mg vs structured lifestyle modification on body weight, liver fat and liver function in obese patients with non-alcoholic fatty liver disease: a pilot randomized trial. Diabetes Obes Metab (2017) 19(12):1814–7. 10.1111/dom.13007 28503750

[22] Khoo J Hsiang JC Taneja R Koo SH Soon GH Kam CJ. Randomized trial comparing effects of weight loss by liraglutide with lifestyle modification in non-alcoholic fatty liver disease. Liver Int (2019) 39(5):941–9. 10.1111/liv.14065 30721572

[23] Liu L Yan H Xia M Zhao L Lv M Zhao N. Efficacy of Exenatide and Insulin Glargine on Nonalcoholic Fatty Liver Disease in Patients with Type 2 Diabetes Mellitus. Diabetes/Metabol Res Rev (2020) 36(5):e3292. 10.1002/dmrr.3292 31955491

[24] Shao N Kuang HY Hao M Gao XY Lin WJ Zou W. Benefits of exenatide on obesity and non-alcoholic fatty liver disease with elevated liver enzymes in patients with type 2 diabetes. Diabetes/Metabol Res Rev (2014) 30(6):521–9. 10.1002/dmrr.2561 24823873

[25] Yan J Yao B Kuang H Yang X Huang Q Hong T. Liraglutide, Sitagliptin, and Insulin Glargine Added to Metformin: The Effect on Body Weight and Intrahepatic Lipid in Patients With Type 2 Diabetes Mellitus and Nonalcoholic Fatty Liver Disease. Hepatology (2019) 69(6):2414–26. 10.1002/hep.30320 PMC6594101 30341767

[26] Zhang LY Qu XN Sun ZY Zhang Y. Effect of liraglutide therapy on serum fetuin A in patients with type 2 diabetes and non-alcoholic fatty liver disease. Clinics Res Hepatol Gastroenterol (2020) 44(5):674–80. 10.1016/j.clinre.2020.01.007 32113823

[27] Astrup A Rössner S Van Gaal L Rissanen A Niskanen L Al Hakim M. Effects of liraglutide in the treatment of obesity: a randomised, double-blind, placebo-controlled study. Lancet (2009) 374(9701):1606–16. 10.1016/S0140-6736(09)61375-1 19853906

[28] Eslam M Sarin SK Wong VW Fan JG Kawaguchi T Ahn SH. The Asian Pacific Association for the Study of the Liver clinical practice guidelines for the diagnosis and management of metabolic associated fatty liver disease. Hepatol Int (2020) 14(16):800–9. 10.1007/s12072-020-10094-2 33006093

[29] Gupta NA Mells J Dunham RM Grakoui A Handy J Saxena NK. Glucagon-like peptide-1 receptor is present on human hepatocytes and has a direct role in decreasing hepatic steatosis in vitro by modulating elements of the insulin signaling pathway. Hepatology (2010) 51(5):1584–92. 10.1002/hep.23569 PMC2862093 20225248

[30] Liu Q Cai BY Zhu LX Xin X Wang X An ZM. Liraglutide modulates gut microbiome and attenuates nonalcoholic fatty liver in db/db mice. Life Sci (2020) 261:118457. 10.1016/j.lfs.2020.118457 32961235

[31] Mells JE Fu PP Sharma S Olson D Cheng L Handy JA. Glp-1 analog, liraglutide, ameliorates hepatic steatosis and cardiac hypertrophy in C57BL/6J mice fed a Western diet. Am J Physiol Gastrointest Liver Physiol (2012) 302(2):G225–35. 10.1152/ajpgi.00274.2011 PMC3341115 22038829

[32] Yang M Ma X Xuan X Deng H Chen Q Yuan L. Liraglutide Attenuates Non-Alcoholic Fatty Liver Disease in Mice by Regulating the Local Renin-Angiotensin System. Front Pharmacol (2020) 11:432. 10.3389/fphar.2020.00432 32322207 PMC7156971

[33] Dong Y Lv Q Li S Wu Y Li L Li J. Efficacy and safety of glucagon-like peptide-1 receptor agonists in non-alcoholic fatty liver disease: A systematic review and meta-analysis. Clin Res Hepatol Gastroenterol (2017) 41(3):284–95. 10.1016/j.clinre.2016.11.009 28065744

[34] Fan S Shi X Yao J Zhong M Feng P. The efficacy of glucagon-like peptide 1 receptor agonists in patients with non-alcoholic fatty liver disease: a systematic review and meta-analysis of randomized controlled trials. Rev Esp Enferm Dig (2020) 112(8):627–35. 10.17235/reed.2020.6392/2019 32496108

[35] Euctr ES. A research study on how semaglutide works in people with fatty liver disease and liver damage. (2019). Available at: http://wwwwhoint/trialsearch/Trial2aspx?TrialID=EUCTR2018-004484-31-ES.

[36] Nct. Investigation of Efficacy and Safety of Three Dose Levels of Subcutaneous Semaglutide Once Daily Versus Placebo in Subjects With Non-alcoholic Steatohepatitis. (2016). Available at: https://clinicaltrialsgov/show/NCT02970942.

[37] Nct. A Study on How Semaglutide Works on Early Stages of Scar Tissue in the Liver Assessed by Pictures of the Liver. (2017). Available at: https://clinicaltrialsgov/show/NCT03357380.

[38] Nct. Researching an Effect of GLP-1 Agonist on Liver STeatosis (REALIST). (2018). Available at: https://clinicaltrialsgov/show/NCT03648554.

[39] Nct. A Research Study on How Semaglutide Works in People With Fatty Liver Disease and Liver Damage. (2019). Available at: https://clinicaltrialsgov/show/NCT03987451.

[40] Nct MSK. Effect of Dulaglutide on Liver Fat in Patients With Type 2 Diabetes and Nonalcoholic Fatty Liver Disease. (2018). Available at: https://clinicaltrialsgov/show/NCT03590626.

[41] Seko Y Sumida Y Tanaka S Mori K Taketani H Ishiba H. Effect of 12-week dulaglutide therapy in Japanese patients with biopsy-proven non-alcoholic fatty liver disease and type 2 diabetes mellitus. Hepatol Res (2016) 47(11):1206–11. 10.1111/hepr.12837 27917557

