# Supplementary material for: Comparison of the Efficacy of Glucagon-Like Peptide-1 Receptor Agonists in Patients With Metabolic Associated Fatty Liver Disease: Updated Systematic Review and Meta-Analysis
Source: Front Endocrinol (Lausanne). 2021 Feb 16;11:622589. doi: 10.3389/fendo.2020.622589 (PMC7924308; doi:10.3389/fendo.2020.622589)
Supplement: Supplementary file 1 [file Table_1.docx]

Supplementary Material

**Supplementary Tables**

**Supplemental Table 1. Search strategies for randomized controlled trails**

| **Database** | **Search strategy** | | **Results** |
| --- | --- | --- | --- |
| **1)PubMed/MEDLINE (To 20 April 2020)** | #1 | Non-alcoholic Fatty Liver Disease[mh] OR Non-alcoholic Fatty Liver Disease*[tiab] OR Nonalcoholic Fatty Liver Disease*[tiab] OR Nonalcoholic Fatty Liver Disease*[tiab] OR NAFLD[tiab] OR Non-alcoholic Steatohepatiti*[tiab] OR Nonalcoholic Steatohepatiti*[tiab] OR Nonalcoholic Steatohepatiti*[tiab] OR Fatty Liver[mh] OR Fatty Liver*[tiab] OR Steatohepatiti*[tiab] OR Steatosis of Liver[tiab]) OR Visceral Steatos*[tiab] OR Liver Steatos*[tiab] | 46084 |
|  | #2 | glucagon-Like Peptide 1[mh] OR "glucagonlike peptide 1"[tiab] OR "GLP 1"[tiab] OR GLP1[tiab] OR liraglutide[mh] OR exenatide[tiab] OR "AC 2993"[tiab] OR "ITCA 650"[tiab] OR liraglutide[tiab]OR "NN 2211"[tiab] OR NN2211[tiab] OR albiglutide[tiab] OR elsiglutide[tiab] OR lixisenatide[tiab] OR "AVE 0010"[tiab] OR dulaglutide[tiab] OR LY2189265[tiab] OR "LY 2189265"[tiab] OR "BIM 51077"[tiab] OR BIM51077[tiab] OR R1583[tiab] OR semaglutide[tiab] OR NN9535[tiab] OR teduglutide[tiab] OR "ALX0600"[tiab] OR ALX0600[tiab] | 15895 |
|  | #3 | ((randomized controlled trail[pt]) OR (controlled clinical trail[pt]) OR randomized[tiab] OR placebo[tiab] OR (clinical trails as topic[mesh:noexp]) OR randomly[tiab] OR trail[ti]) NOT (animal[mh] NOT humans[mh]) | 797251 |
|  | #4 | #1 AND #2 AND #3 | 56 |
| **2) EMBASE**  **(To 20 April 2020)** | #1 | 'nonalcoholic fatty liver'/exp OR 'nonalcoholic steatohepatitis'/exp OR ''non-alcoholic fatty liver*':ti,ab,kw OR 'nonalcoholic fatty liver*':ti,ab,kw OR nafld:ti,ab,kw OR 'non-alcoholic steatohepatiti*':ti,ab,kw OR 'nonalcoholic steatohepatiti*':ti,ab,kw OR 'fatty liver'/exp OR 'steatohepatitis'/exp OR 'fatty liver*':ti,ab,kw OR steatohepatiti*:ti,ab,kw OR 'steatosis of liver':ti,ab,kw OR 'visceral steatos*':ti,ab,kw OR 'liver steatos':ti,ab,kw | 85506 |
|  | #2 | 'glucagon like peptide 1 receptor agonist'/exp OR 'glucagon like peptide 1 receptor agonist*':ti,ab,kw OR 'glucagon like peptide 1 receptor analog*':ti,ab,kw OR 'glp-1 agonist*':ti,ab,kw OR 'glp-1 analog*':ti,ab,kw OR (exenatide or AC 2993 or ITCA 650).tw. OR (liraglutide or NN 2211 or NN2211 or NNC 90 1170 or NNC90 1170).tw. OR (albiglutide or GSK 716155).tw. OR (elsiglutide).tw. OR (lixisenatide or AVE 0010).tw. OR (dulaglutide or LY2189265 or LY 2189265).tw. OR (taspoglutide or BIM 51077 or BIM51077 or ITM 077 or ITM077 or R 1583 or R1583 or RO 5073031 or RO5073031).tw. OR (semaglutide or NN 9535 or NN9535).tw. OR (teduglutide or ALX 0600 or ALX0600).tw. | 33728 |
|  | #3 | 'crossover-procedure'/exp OR 'double-blind procedure'/exp OR 'randomized controlled trial'/exp OR 'single-blind procedure'/exp OR ((((random* OR factorial* OR crossover* OR cross) AND over* OR 'cross over*' OR placebo* OR double*) AND adj AND blind* OR single*) AND adj AND blind*) OR assign* OR allocate* OR volunteer*.af | 1014953 |
|  | #4 | #1 AND #2 AND #3 | 92 |
| **3) Cochrane Central Register of Controlled Trials**  **(To 20 April 2020)** | #1 | MeSH descriptor: [Non-alcoholic Fatty Liver Disease] explode all trees OR (Non-alcoholic Steatohepatiti*):ti,ab,kw OR (Nonalcoholic Steatohepatiti*):ti,ab,kw OR (Nonalcoholic Steatohepatiti*):ti,ab,kw OR (Non-alcoholic Fatty Liver*):ti,ab,kw OR (Nonalcoholic Fatty Liver*):ti,ab,kw OR (Nonalcoholic Fatty Liver*):ti,ab,kw AND (NAFLD):ti,ab,kw OR MeSH descriptor: [Fatty Liver] explode all trees OR ("fatty liver"):ti,ab,kw OR (Steatohepatiti*):ti,ab,kw OR ("Steatosis of Liver"):ti,ab,kw OR (Visceral Steatos*):ti,ab,kw OR (Liver Steatos*):ti,ab,kw | 3576 |
|  | #2 | MeSH descriptor: [Glucagon-Like Peptide 1] explode all trees OR ((glucagon like peptide* or GLP 1 or GLP1) adj3 (analog* or agonist*)):ti,ab,kw OR (exenatide or AC 2993 or ITCA 650):ti,ab,kw OR (liraglutide or NN 2211 or NN2211 or NNC 90 1170 or NNC90 1170):ti,ab,kw OR (albiglutide or GSK 716155):ti,ab,kw OR (elsiglutide):ti,ab,kw OR (lixisenatide or AVE 0010):ti,ab,kw OR (dulaglutide or LY2189265 or LY 2189265):ti,ab,kw OR (taspoglutide or "BIM 51077" or BIM51077 or ITM 077 or ITM077 or R 1583 or R1583 or RO 5073031 or RO5073031):ti,ab,kw OR (semaglutide or NN 9535 or NN9535):ti,ab,kw OR (teduglutide or ALX 0600 or ALX0600):ti,ab,kw | 4627 |
|  | #3 | #1 AND #2 | 105 |
| **4) Web of science (To 20 April 2020)** | #1 | TS = ((liver and (fatty or steatosis or steatoses)) or NAFLD or NASH) | 115084 |
|  | #2 | TS = (((glucagon-Like Peptide 1 or glucagon like peptide 1 or GLP-1) and ((receptor agonist) or (receptor agonism))) or (liraglutide or "NN 2211" or NN2211) or (exenatide or "AC 2993" or "ITCA 650") or (albiglutide) or (elsiglutide) or (lixisenatide or "AVE 0010") or (dulaglutide or LY2189265 or "LY 2189265"or "BIM 51077" or BIM51077 or R1583) or (semaglutide or NN9535) or (teduglutide or "ALX0600" or ALX0600)) | 11791 |
|  | #3 | TS= ((random* OR rct* OR crossover OR masked OR blind* OR placebo*) NOT (animal)) | 2325269 |
|  | #4 | #1 AND #2 AND #3 | 113 |

**Supplemental Table 2. Baseline characteristic of participants in the eight included studies**

| Author | LFF/LFC (%) | | Body weight (kg) | | Waist circumference (cm) | | ALT (U/L) | |
| --- | --- | --- | --- | --- | --- | --- | --- | --- |
|  | GLP-RAs | Control | GLP-RAs | Control | GLP-RAs | Control | GLP-RAs | Control |
| Armstrong | NR | NR | 101(18) | 108(18) | 110(11) | 120(15) | 77(34) | 66(42) |
| Feng W | 36.70(3.65) | 32.99(3.51) | 81.10(2.27) | 79.71(2.63) | 95.60(1.42) | 96.69(1.77) | 49.73(5.79) | 44.99(5.07) |
| Khoo J. 2017 | 28.3(9.3) | 32.1(17.9) | 98.0(14.5) | 90.1(7.9) | 108.0(6.4) | 106.0(7.9) | 87(38) | 98(38) |
| Khoo J. 2019 | 31.4(9.3) | 30.8(17.5) | 102.7(16.2) | 89.6(12.7) | 111.1(10.7) | 105.8(7.6) | 87(32) | 88(38) |
| Liu L. | 42.12(16.83) | 35.47(13.78) | 79.28(9.64) | 77.63(13.70) | 97.17(9.35) | 97.33(9.20) | 42.71(23.19) | 32.81(22.37) |
| Shao N. | NR^1^ | NR^1^ | 86.36(3.09) | 85.49(2.70) | 101.94(2.30) | 100.82(2.28) | 169.54(18.23) | 163.93(22.97) |
| Yan J. | 15.4(5.6) | 14.9(5.5) | 86.6(12.9) | 85.6(14.2) | 101.7(7.9) | 102.9(9.9) | 43.2(21.2) | 39.5(25.7) |
| Zhang L. | 24.1(3.0) | 23.9(3.8) | 79.3(8.8) | 78.0(9.2) | 93.2(4.6) | 91.6(7.2) | 3.4(0.3) ^*^ | 3.6(0.5) ^*^ |

| Author | AST (U/L) | | γ-GGT (U/L) | | FBG (mmol/L) | | HbA1c (%) | |
| --- | --- | --- | --- | --- | --- | --- | --- | --- |
|  | GLP-RAs | Control | GLP-RAs | Control | GLP-RAs | Control | GLP-RAs | Control |
| Armstrong | 51(22) | 51(27) | 91(69) | 115(174) | 6.0(1.7) | 6.1(1.5) | 5.9(0.7) | 6.0(0.9) |
| Feng W | 31.22(2.56) | 28.45(2.47) | NR | NR | 8.80(0.44) | 8.96(0.30) | 8.91(0.32) | 9.03(0.23) |
| Khoo J. 2017 | 49(19) | 56(27) | NR | NR | 5.7(0.6) | 6.2(1.1) | NR | NR |
| Khoo J. 2019 | 45(14) | 52(27) | NR | NR | 5.7(0.5) | 6.1(0.8) | NR | NR |
| Liu L. | 31.29(17.32) | 25.11(14.09) | 55.40(43.35) | 63.03(64.97) | 9.20(1.66) | 9.69(2.39) | 8.32(0.94) | 8.58(0.91) |
| Shao N. | 125.18(16.38) | 121.86(15.58) | 136.07(9.94) | 135.26(7.41) | 9.35(1.14) | 9.19(1.14) | 7.68(0.57) | 7.59(0.57) |
| Yan J. | 31.1(11.7) | 33.2(17.4) | NR | NR | 8.6(2.8) | 8.9(2.2) | 7.8(1.4) | 7.7(0.9) |
| Zhang L. | 3.5(0.3) ^*^ | 3.5(0.4) ^*^ | 3.9(0.4) ^*^ | 3.7(0.5) ^*^ | 8.9(2.9) | 8.7(3.3) | 8.1(2.0) | 8.1(1.7) |

Data are the mean (standard deviation). GLP-1RAs, glucagon-like peptide-1 receptor agonists; ALT, alanine aminotransferase; AST, aspartate aminotransferase; FBG, fasting blood-glucose; γ-GGT, gamma-glutamyl transpeptidase; LFC, liver fat content; LFF, liver fat fraction; NR, not reported.

^1^ Repressed as hierarchical data.

^*^ Natural logarithms.
